# HIV and HCV: distinct infections with important overlapping challenges

**DOI:** 10.7448/IAS.17.1.19323

**Published:** 2014-07-28

**Authors:** Stefan Wiktor, Nathan Ford, Andrew Ball, Gottfried Hirnschall

**Affiliations:** HIV/AIDS Department, World Health Organization, Geneva, Switzerland

## Introduction

Up to one in seven people living with HIV are co-infected with hepatitis C virus (HCV) [1], and HCV is now a leading cause of death among people living with HIV in some countries [2]. HCV and HIV have a number of important overlapping challenges. While the vast majority of the estimated 180 million people infected with HCV globally are HIV negative, efforts to control HCV infection can be informed by the experience of the global HIV response. Many of the key challenges for scaling up access to HCV treatment have had to be confronted for HIV, including ensuring access to medicines, developing simplified models of care, scaling up prevention alongside treatment, reaching marginalized populations, overcoming stigma and supporting civil society.

## Ensuring affordable access to treatment

The annual cost of HIV treatment initially was around $US10,000 per patient [3], but is now available at around $US120 in some countries, and worldwide almost 13 million persons were receiving ART as of the end of 2013. This price reduction and scale-up was the result of concerted efforts by national governments, international organizations, civil society and the pharmaceutical industry to make quality treatment affordable in resource-limited settings. Today, efforts to scale-up anti-HCV therapy face similar challenges. After decades of poor treatment outcomes using drugs discovered over 40 years ago (ribavirin [4] and interferon [5]), a robust drug development pipeline has begun to deliver a number of safe and highly effective treatments; however, the cost of these drugs is currently too high to allow for widespread treatment scale-up, even in high-income settings [6]. Lack of access to treatment also contributes to the low levels of HCV diagnosis in many settings, a dynamic also seen in the early days of the HIV epidemic where patients and health providers were unmotivated to test for HIV in the absence of treatment [7].

Ensuring affordable access to these new drugs will be a precondition for scaling-up HCV treatment globally. Comprehensive strategies that helped improve access to affordable HIV medicines and diagnostics included voluntary and compulsory licensing, patent-sharing arrangements and improved procurement mechanisms which can, in principle, be replicated for HCV. Pharmaceutical companies, international organizations, procurement and funding mechanisms, national governments and civil society all need to play a part in ensuring access to affordable treatment for HCV.

## Developing simplified models of care

Over the last decade, continuous efforts have been made to simplify HIV treatment regimens, patient monitoring and models of service delivery in order to support increased access to treatment and care in resource-limited settings [8]. Initially, a specialized infectious disease approach was used to manage people living with HIV. Today, in many settings the majority of people receiving antiretroviral therapy are managed by nurses in primary care settings; laboratory and treatment requirements have been reduced to a minimal essential package, and high-quality research has validated the safety of shifting tasks from specialists and doctors to non-physician providers [9]. Responding to HCV at scale will require a similar approach. Pilot programmes in high-income settings have already begun to assess approaches to task shifting and decentralization of HCV care [10,11], and such approaches will be even more important in settings where health resources are scarce.

## Scaling up prevention alongside treatment

Common routes of HCV transmission include injecting drug use, unsafe medical injections and other invasive procedures, contaminated blood products, and less common routes of transmission include mother-to-child transmission, sexual transmission (particularly among HIV-positive individuals), and non-sterile tattoo or piercing procedures. Most of these are also common routes of transmission for HIV and as such the scale-up of prevention services for HIV can reduce the incidence of HCV infection, and vice versa. Improving access to key prevention interventions is in many countries limited by weak blood safety and infection control systems and policies that restrict access to sterile equipment for drug injecting, tattooing and skin piercing, notably in closed settings such as prisons, along with suboptimal availability of condoms, particularly for sex workers and their clients and men who have sex with men. The increased recognition of HCV as a global infectious disease priority provides an opportunity to improve access to basic prevention interventions that can significantly impact on both HCV and HIV transmission. Several vaccines for both HIV and HCV are under development, but none have elicited a level of efficacy to be able to contribute to prevention efforts.

## Overcoming stigma

HIV and HCV are both stigmatized infections in many societies. In addition, people who are at heightened risk of becoming infected with either or both of these viruses – people who inject drugs, sex workers, men who have sex with men, HIV-positive individuals, and prisoners – are often among the most marginalized in society, with very limited access to health services. Efforts to reduce HIV incidence and mortality have been most successful in countries that have recognized the need to address stigma and ensure access to services for all. Stigma reduction is therefore another critical area that will benefit both HIV and HCV control activities. One obvious example where improved services for marginalized populations will support reductions in incidence of both HIV and HCV is expanded access to safe injecting equipment and opioid substitution therapy for people who inject drugs.

## Supporting civil society

Civil society activism has been critical to lobbying for improved access to affordable treatment, mobilizing and maintaining political action and financial resources against HIV, highlighting gaps in the response to HIV both globally and locally, and protesting against policies that may limit the rights of people living with HIV. Similar activism is already contributing to improved access to treatment and care for people with HCV, including from groups working on HIV. However, much more political and financial support is needed to strengthen the HCV activism movement.

## Conclusions

HCV is an epidemic of global importance, and only a minority of cases are associated with HIV. Nevertheless, the experience gained in responding to HIV provides important opportunities to improve prevention, treatment and care services for people infected with and affected by HCV, strengthening health information systems and encouraging the uptake of policies and improving access to effective, affordable treatment. At the same time, the recognition of the scale of the HCV epidemic has reinforced the need to strengthen blood and injection safety programmes and to improve cancer screening and management services, also important for HIV responses. Over the coming years, the World Health Organization will work to promote policy and programme synergies in the global and national response against both diseases.
